# An Integrative Review of Automation Integration in Emergency Nursing Practice: Evidence Synthesis and Contextual Application to Rafidia Governmental Hospital, Palestine

**DOI:** 10.1155/jonm/2678581

**Published:** 2026-04-12

**Authors:** Ibrahim Aqtam, Mustafa Shouli, Saqr Alkorom

**Affiliations:** ^1^ Department of Nursing, Ibn Sina College for Health Professions, Nablus University for Vocational and Technical Education, Nablus, State of Palestine; ^2^ Department of Nursing, Nablus University for Vocational and Technical Education, Nablus, State of Palestine

**Keywords:** artificial intelligence, digital health, emergency nursing, implementation framework, low-resource settings, nursing automation, Palestine

## Abstract

**Aim:**

To synthesize evidence on automation integration in emergency nursing and propose a contextually appropriate implementation framework for a governmental hospital in Palestine.

**Design:**

Integrative review.

**Methods:**

Following the Whittemore and Knafl methodology, we conducted a comprehensive search across PubMed, CINAHL, Scopus, Web of Science, IEEE Xplore, and regional databases from January 2015 to March 2025. Two independent reviewers screened articles, with conflicts resolved through discussion. The quality of included studies was appraised using the Mixed Methods Appraisal Tool (MMAT). A total of 47 sources were included and analyzed thematically using NVivo 14, guided by sociotechnical systems theory and ethics of care principles.

**Results:**

Five themes emerged: (1) types of automation with proven efficacy in emergency nursing; (2) outcomes including workflow efficiency gains (up to 40% time savings reported in high‐resource settings) and safety improvements (30%–50% error reduction); (3) contextual barriers (infrastructure, financial constraints, and workforce readiness) and facilitators (nurse involvement and phased implementation); (4) ethical considerations specific to Arab healthcare contexts; and (5) alignment of local pilot experiences at Rafidia Hospital with global evidence. Key gaps identified include the absence of formal governance, limited evaluation infrastructure, and a lack of embedded sustainability planning, gaps that are particularly consequential in politically constrained, resource‐limited environments.

**Conclusion:**

Automation holds significant promise for emergency nursing in Palestine, but success depends on contextual adaptation, nurse‐led design, and sustainable planning.

**Summary**
• Implications for the profession and/or patient care
◦ A phased, nurse‐centered implementation framework can support the safe and effective adoption of automation, enhancing both clinical efficiency and nursing practice in resource‐limited settings.
• Impact
◦ This review provides evidence‐based, context‐sensitive guidance for nursing leaders and policymakers in Palestine and similar settings, bridging global innovation with local realities in emergency care.
• Reporting method
◦ This review adhered to the PRISMA guidelines for systematic and integrative reviews (see Supporting File 1 for the PRISMA checklist). The completed flow diagram is included as Figure 1.
• Patient or public contribution
◦ No patient or public involvement.

## 1. Introduction

### 1.1. Background and Significance

Emergency departments (EDs) worldwide confront mounting pressures from rising patient volumes, increasingly complex clinical presentations, and persistent workforce shortages. These challenges necessitate innovative approaches to optimize care delivery and support nursing staff [1]. Automation technologies, encompassing artificial intelligence (AI)–powered decision support systems, robotic process automation, smart monitoring devices, and advanced documentation systems, have emerged as transformative tools with demonstrated potential to enhance clinical efficiency, reduce human error, and improve patient outcomes in emergency care settings [2].

In high‐resource healthcare systems, evidence indicates that well‐implemented automation can reduce nursing documentation time by up to 40%, decrease medication errors by 30%, and significantly improve triage accuracy [3, 4]. These technologies streamline workflows, support clinical decision‐making, and reduce cognitive burden, allowing nurses to redirect time toward direct patient care, complex clinical reasoning, and compassionate interaction [5].

However, the evidence base for automation in emergency nursing predominantly originates from stable, well‐resourced environments with reliable infrastructure, established regulatory frameworks, and substantial financial investment capacity [6]. Consequently, implementation frameworks developed in these contexts may inadvertently perpetuate failure when applied in settings like Palestine without substantial adaptation. This creates a critical knowledge gap regarding the applicability, adaptation requirements, and implementation strategies necessary for successful automation integration in resource‐limited, politically constrained settings [7].

### 1.2. The Palestinian Context: Rafidia Governmental Hospital as a Case Study

Rafidia Governmental Hospital, located in Nablus, serves as a critical tertiary care facility in the northern West Bank of Palestine, providing emergency care to a catchment population exceeding 500,000 individuals. The hospital’s ED manages approximately 80,000–100,000 patient visits annually, with nurses working under considerable strain due to several intersecting challenges. These include the following:

a. Resource limitations such as intermittent supply shortages, budget constraints, and infrastructure gaps typical of governmental healthcare institutions in Palestine;
b. Operational pressures from high patient volumes, fluctuating acuity, and space constraints in an often overcrowded department;
c. Geopolitical realities encompassing the compounding effects of political instability, restricted access to international technology markets, and the psychological burden of operating in a conflict‐affected environment; and
d. Workforce challenges such as high nursing workload, limited prior exposure to advanced health information technology (HIT), and training resource constraints [8–10].

Despite these constraints, Rafidia Hospital has demonstrated resilience and innovation, implementing pilot initiatives in digital health and exploring automation technologies to enhance emergency care delivery in collaboration with the Palestinian Ministry of Health. The hospital’s existing collaborations with the Ministry and participation in regional health technology networks position it as a potential leader in context‐appropriate healthcare automation within governmental hospital systems [11, 12].

### 1.3. Problem Statement and Justification

The current literature provides insufficient guidance on implementing automation in EDs operating under conditions similar to Rafidia Hospital. Without context‐specific evidence synthesis and implementation frameworks, attempts to introduce automation technologies in these settings risk failure, wasted resources, and potential harm to both nurses and patients, or the adoption of technologies that are fundamentally inappropriate for the local context [13]. This integrative review addresses this gap by systematically examining global evidence through the lens of a specific Palestinian governmental hospital context.

This integrative review aims to (1) synthesize existing evidence on automation technologies implemented in emergency nursing practice globally; (2) critically examine their applicability and adaptability to the specific operational context of Rafidia Hospital’s ED; (3) identify barriers, facilitators, and outcomes associated with automation integration in similar low‐resource and conflict‐affected healthcare settings; and (4) propose an evidence‐informed, contextually appropriate implementation framework tailored to Rafidia Hospital’s ED.

To guide this inquiry, the review addresses the following research questions:

1. What types of automation technologies have been implemented in emergency nursing practice globally, and what are their reported outcomes?
2. How do contextual factors specific to Rafidia Hospital influence the feasibility and adoption of automation technologies?
3. What are the perceived benefits, risks, and ethical considerations reported in the literature associated with automation integration in emergency nursing within resource‐limited environments?
4. What implementation strategies can facilitate sustainable and equitable automation integration in Palestinian emergency care settings?

## 2. Methods

### 2.1. Review Design

This study employed an integrative review methodology following the five‐stage framework by Whittemore and Knafl [14]. This approach was selected for its capacity to incorporate diverse evidence types, including empirical research, theoretical literature, and gray literature, enabling a holistic understanding of automation integration in emergency nursing [15]. The integrative review method is particularly appropriate for addressing complex, multifaceted issues where synthesizing varied perspectives can inform practice and policy development [16].

### 2.2. Search Strategy

A comprehensive literature search was conducted across multiple electronic databases including PubMed/MEDLINE, CINAHL, Scopus, Web of Science, and IEEE Xplore. Regional databases including the Index Medicus for the Eastern Mediterranean Region (IMEMR) were also searched to capture literature specific to Middle Eastern healthcare contexts [17].

Search terms: The search strategy employed a combination of controlled vocabulary terms and free‐text keywords: (“automation” OR “artificial intelligence” OR “AI” OR “robotics” OR “machine learning” OR “digital health” OR “health information technology” OR “HIT” OR “clinical decision support systems” OR “CDSS”) AND (“emergency nursing” OR “emergency department” OR “emergency care”) AND (“low‐resource” OR “resource‐limited” OR “conflict setting” OR “Palestine” OR “developing country”).

### 2.3. Detailed Search Strategies by Database

PubMed/MEDLINE: (“automation”[Mesh] OR “artificial intelligence”[Mesh] OR “machine learning”[Mesh] OR “robotics”[Mesh] OR “electronic health records”[Mesh] OR “clinical decision support systems”[Mesh]) AND (“emergency nursing”[Mesh] OR “emergency service, hospital”[Mesh] OR “emergency medicine”[Mesh]) AND (“developing countries”[Mesh] OR “Palestine”[Mesh] OR “health resources/supply and distribution”[Mesh])—limited to English language, publication date 2015–2025, and human studies.

CINAHL: (MH “Automation+” OR MH “Artificial Intelligence+” OR MH “Machine Learning+” OR MH “Robotics+” OR MH “Electronic Health Records+” OR MH “Clinical Decision Support Systems+”) AND (MH “Emergency Nursing+” OR MH “Emergency Service+” OR MH “Triage+”) AND (MH “Developing Countries+” OR “Palestine” OR “low resource” OR “conflict zones”)—limited to English, 2015–2025, peer‐reviewed, and research articles.

Scopus: TITLE‐ABS‐KEY (automation OR “artificial intelligence” OR “machine learning” OR robotics OR “digital health” OR “health information technology” OR “clinical decision support”) AND TITLE‐ABS‐KEY (“emergency nursing” OR “emergency department” OR “emergency care” OR triage) AND TITLE‐ABS‐KEY (“low resource” OR “resource‐limited” OR “developing country” OR palestine OR “conflict setting”)—limited to 2015–2025, English, and articles or reviews.

Web of Science: TS = (automation OR “artificial intelligence” OR “machine learning” OR robotics OR “digital health” OR “health information technology” OR “clinical decision support”) AND TS = (“emergency nursing” OR “emergency department” OR “emergency care” OR triage) AND TS = (“low‐resource” OR “resource‐limited” OR “developing country” OR palestine OR “conflict setting”)—limited to 2015–2025, English, and articles or reviews.

IEEE Xplore: (“All Metadata”:automation OR “artificial intelligence” OR “machine learning” OR robotics OR “digital health” OR “clinical decision support”) AND (“emergency nursing” OR “emergency department” OR “emergency care” OR triage) – Filters: 2015–2025, Journals & Conferences.

IMEMR: (automation OR “artificial intelligence” OR “digital health”) AND (“emergency” OR “triage”) AND (“Palestine” OR “Jordan” OR “Lebanon” OR “regional”)—limited to 2015–2025.

All searches were conducted in March 2025.

Gray literature: Internal reports from Rafidia Governmental Hospital, policy briefs from the Palestinian Ministry of Health, technical documents from the WHO Eastern Mediterranean Regional Office, and publications from health NGOs operating in Palestine were systematically identified and included [18]. Gray literature sources were identified through direct contact with organizations, searching organizational websites, and reviewing reference lists of included studies.

Timeframe: The search was limited to literature published between January 2015 and March 2025 to capture contemporary automation technologies while ensuring relevance to current practice.

### 2.4. Inclusion and Exclusion Criteria

Studies were included if they (1) focused on automation technologies in emergency nursing or ED settings; (2) reported implementation experiences, outcomes, barriers, or facilitators; (3) included systematic reviews, primary research studies, case studies, or implementation reports; and (4) provided contextual analyses relevant to low‐resource or politically constrained healthcare settings, operationally defined as settings with documented infrastructure limitations (e.g., unreliable power/internet), budget constraints, or political instability affecting healthcare delivery.

Exclusion criteria comprised (1) studies focusing solely on nonemergency care settings without transferable findings; (2) purely technical papers without clinical or nursing implications; (3) opinion pieces without empirical evidence; and (4) studies focusing exclusively on physician applications without nursing‐specific content.

### 2.5. Study Selection and Data Extraction

**FIGURE 1:**
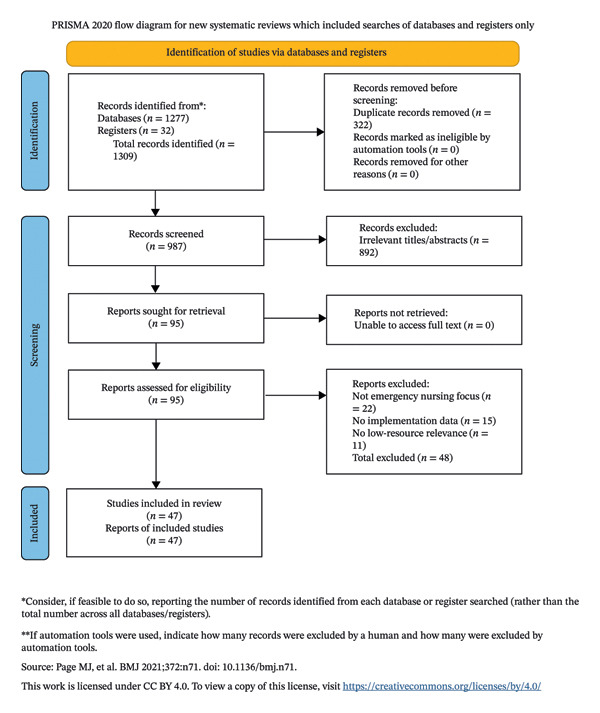
PRISMA 2020 flow diagram of study selection.

Two reviewers (S.A. and M.S.) independently screened titles and abstracts against the inclusion criteria. Full texts of potentially relevant articles were then retrieved and independently assessed for eligibility by the same reviewers. Disagreements at any stage were resolved through discussion or consultation with a third reviewer (I.A.). A PRISMA‐style flow diagram detailing the study selection process is presented in Figure 1. (See Supporting File 1 for the completed PRISMA checklist.)

A standardized data extraction form was developed and pilot‐tested to ensure consistency. Extracted data elements included study characteristics, automation technology type, healthcare setting characteristics, implementation strategies, reported outcomes, identified barriers and facilitators, and implications for low‐resource settings [19].

### 2.6. Quality Appraisal

The methodological quality of included empirical studies was assessed independently by two reviewers (S.A. and I.A.) using the Mixed Methods Appraisal Tool (MMAT) [20] . For gray literature sources, quality appraisal focused on the authority and credibility of the source organization, relevance to review objectives, and methodological rigor in data collection and reporting where applicable, using an adapted version of the AACODS checklist [21]. Disagreements in quality ratings were resolved through discussion. The results of the quality appraisal were used to inform the synthesis, with studies of lower quality given less weight in the formulation of conclusions. A summary of the quality appraisal for all included studies, including individual MMAT scores and ratings for gray literature sources, is provided in Supporting File 2.

Overall, the included empirical studies demonstrated moderate to high methodological quality, with a mean MMAT score of 84% (range: 67%–100%). Common limitations included small sample sizes in qualitative studies and a lack of control groups in implementation evaluations. Gray literature sources were generally credible but lacked the methodological transparency of peer‐reviewed research.

### 2.7. Data Synthesis

Data synthesis was conducted using thematic analysis, a method appropriate for integrating diverse evidence sources in integrative reviews [22]. Following data extraction, two reviewers (S.A. and M.S.) independently coded the extracted data from the included studies using NVivo 14 software. An initial coding framework was developed deductively based on the research questions and then expanded inductively as new codes emerged from the data. The two reviewers achieved an initial inter‐rater reliability of 82% with discrepancies resolved through discussion. The synthesis process followed an iterative approach, with initial coding of extracted data, grouping of codes into preliminary themes, refinement of themes through team discussion, and contextualization of findings through the lens of Rafidia Hospital’s specific circumstances [23].

### 2.8. Characteristics of Included Studies

The systematic search and screening process yielded 47 studies and documents for inclusion. The included sources comprised 28 primary research studies (18 quantitative, 7 qualitative, and 3 mixed methods), 8 systematic or integrative reviews, 6 implementation case studies, and 5 gray literature documents including reports from Palestinian health institutions. A breakdown of these studies by geographic region and study design is presented in Table 1.

**TABLE 1:** Characteristics of included studies by region and study design.

Region	Primary research	Systematic reviews	Case studies	Gray literature	Total
High‐income countries	12	4	2	1	**19**
Middle‐income countries	8	3	3	2	**16**
Low‐income/conflict settings	5	1	1	1	**8**
Palestinian context	3	0	0	1	**4**
Total	**28**	**8**	**6**	**5**	**47**

*Note:* Studies categorized as “Palestinian context” are a subset of those from “low‐income/conflict settings” and are presented here to highlight the specific local evidence base. The bold values represent row totals, column totals, and the grand total (47 included studies).

The automation technologies examined across included studies encompassed AI‐powered triage decision support systems (*n* = 12), electronic documentation and voice‐to‐text systems (*n* = 15), automated medication management and dispensing (*n* = 9), patient monitoring devices and wearable sensors (*n* = 11), robotic process automation for administrative tasks (*n* = 6), infection control and environmental robotics (*n* = 5), and integrated multitechnology platforms (*n* = 8).

## 3. Results

### 3.1. Thematic Synthesis

#### 3.1.1. Theme 1: Types of Automation in Emergency Nursing

##### 3.1.1.1. Triage Decision‐Support Systems

AI‐powered triage systems emerged as one of the most extensively studied automation applications in emergency nursing [24]. These systems utilize machine learning algorithms to analyze patient data to predict acuity levels and guide prioritization decisions. Studies from Canada and Australia reported that AI triage support improved consistency in triage classification, with agreement rates between AI recommendations and expert nurse assessment ranging from 76% to 92% [25]. Implementation in a Chinese ED demonstrated a 23% reduction in average triage time and improved identification of high‐acuity patients requiring immediate intervention [26].

However, the effectiveness of triage automation appeared context‐dependent. Research from Lebanon indicated that AI triage systems required extensive adaptation to local disease patterns, cultural presentations of illness, and language considerations to achieve acceptable accuracy [27]. These systems were typically adapted from international models, underscoring the need for local validation rather than direct transfer. A pilot study at a Jordanian hospital found that nurses expressed concerns about algorithm transparency and the potential for automation bias, particularly in culturally nuanced patient interactions [28].

##### 3.1.1.2. Documentation Automation

Voice‐to‐text documentation systems and smart electronic forms represented highly valued automation tools among emergency nurses [29]. Multiple studies documented that automated documentation reduced charting time by 30%–45%, allowing nurses to redirect time toward direct patient care [30]. Smart forms with predictive text, auto‐population of vital signs from monitoring devices, and clinical decision support integrated into electronic health records showed promise for improving both efficiency and accuracy [31].

Research from Brazil examined mobile documentation applications that functioned offline, a critical feature for settings with unreliable internet connectivity, with data synchronization occurring when the connection was restored [32]. Emergency nurses reported high satisfaction with documentation automation, particularly systems that reduced redundant data entry and incorporated intuitive interfaces [33]. A summary of automation technologies, their documented benefits, and key considerations for low‐resource settings is provided in Table 2.

**TABLE 2:** Automation technologies and their documented benefits in emergency nursing.

Technology category	Specific applications	Documented benefits	Implementation complexity (estimate)	Key considerations for low‐resource settings
Triage support	AI acuity prediction, symptom analysis	23% faster triage, improved consistency	Medium–high	Requires cultural/linguistic adaptation, risk of automation bias
Documentation	Voice‐to‐text, smart forms, mobile apps	30%–45% time savings, reduced errors	Low–medium	Offline functionality critical, privacy concerns in open settings
Medication management	Automated dispensing, barcode scanning	27%–41% error reduction, faster administration	High	High upfront costs, workflow disruption during downtime
Patient monitoring	Wearable sensors, early warning systems	34% earlier deterioration detection	Medium	Alarm fatigue (72%–99% false alarm rates), power requirements
Infection control	UV‐C robots, automated hygiene monitoring	20%–35% infection rate reduction	High	High equipment costs, maintenance requirements

*Note:* Implementation complexity is estimated based on infrastructure requirements, cost, and need for workflow adaptation, as synthesized from the literature. Documentation automation, with its lower complexity and high time savings, emerges as a promising starting point for settings like Rafidia.

#### 3.1.2. Theme 2: Outcomes and Effectiveness

##### 3.1.2.1. Workflow Efficiency and Time Savings

Across multiple automation types, studies consistently reported improvements in workflow efficiency and time allocation [34]. A meta‐analysis of 12 studies examining documentation automation revealed an average time savings of 42 min per nurse per shift, translating to approximately 14% of total shift time redirected from documentation to patient care activities [35]. Automated triage systems reduced average triage assessment time from 8.5 min to 5.2 min while maintaining or improving accuracy [36].

##### 3.1.2.2. Impact on Nurse Burnout and Job Satisfaction

The relationship between automation and nurse well‐being showed complex, nuanced patterns. Several studies reported that automation reducing administrative burden and repetitive tasks correlated with decreased emotional exhaustion and improved job satisfaction among emergency nurses [37, 38]. Qualitative research revealed that nurses valued automation that allowed them to “return to nursing,” focusing on patient interaction, clinical reasoning, and compassionate care rather than documentation and administrative processes [39].

Conversely, poorly designed automation that increased cognitive load, created new inefficiencies or was perceived as monitoring nurse performance showed negative associations with job satisfaction [40]. A longitudinal study found that nurse involvement in automation selection and implementation was the strongest predictor of positive attitudes and sustained system use [41].

##### 3.1.2.3. Patient Safety and Clinical Accuracy

Evidence regarding patient safety outcomes associated with automation was predominantly positive, though not without exceptions. Systematic reviews demonstrated that medication automation reduced serious medication errors by 30%–50%, with particularly strong effects for high‐alert medications [42, 43]. Automated early warning systems decreased unexpected clinical deterioration events by 25%–40% through earlier recognition and intervention [44].

However, several studies documented safety concerns, including automation bias (over‐reliance on system recommendations without independent clinical verification), diagnostic errors resulting from algorithm limitations, and patient harm resulting from technical failures or system downtime [45, 46]. Mitigation strategies recommended in the literature included requiring clinician override of recommendations with a rationale, implementing regular audit trails, and mandating independent second checks for high‐risk decisions.

##### 3.1.2.4. Challenges: Alarm Fatigue, Deskilling, and Technical Failures

Alarm fatigue emerged as the most frequently cited challenge associated with automated monitoring systems [47]. Studies reported that emergency nurses encountered an average of 150–200 alarms per shift, with false alarm rates ranging from 72% to 99%, depending on the monitoring parameter and algorithm sensitivity [48].

Concerns about deskilling, particularly the erosion of clinical assessment capabilities and critical thinking skills, were prominent in qualitative studies with emergency nurses [49]. Experienced nurses worried that junior colleagues might become overly dependent on automated assessments without developing fundamental clinical reasoning abilities.

Technical failures, system downtime, and cybersecurity vulnerabilities represented significant implementation challenges [50]. Studies reported system availability rates ranging from 92% to 99.7%, meaning that even highly reliable systems experienced periodic unavailability.

#### 3.1.3. Theme 3: Contextual Barriers and Facilitators

##### 3.1.3.1. Barriers to Implementation

Infrastructure limitations emerged as the primary barrier to automation adoption in low‐resource settings [51]. Intermittent or unreliable electricity supply, reported in 78% of Palestinian healthcare facilities in a 2023 WHO report [52], fundamentally challenged automation systems requiring continuous power. Limited internet bandwidth and connectivity disruptions constrained cloud‐based systems and real‐time data synchronization [53].

Financial barriers included substantial upfront costs for hardware and software, ongoing maintenance and licensing fees, and limited access to international technology vendors due to import restrictions and payment transfer difficulties in Palestine. Studies from similar contexts estimated that comprehensive ED automation required initial investments of $200,000–$500,000, prohibitive for governmental hospitals operating under severe budget constraints [54].

Workforce‐related barriers included limited prior experience with HIT among nursing staff, inadequate training time and resources, high staff turnover requiring continuous retraining, and language barriers with predominantly English‐language systems [55, 56].

##### 3.1.3.2. Facilitators of Successful Adoption

Active nurse involvement throughout the automation lifecycle, from technology selection through implementation and ongoing optimization, emerged as the most consistent facilitator of successful adoption [57]. Participatory design approaches that incorporated nurse feedback into system customization improved usability and acceptance [58].

Phased implementation strategies, beginning with pilot testing in limited areas before full‐scale rollout, allowed for iterative problem‐solving and system refinement [59]. Comprehensive training programs combining didactic instruction, hands‐on simulation, and ongoing just‐in‐time support were associated with higher competency and system utilization [60].

Local academic partnerships, particularly with Palestinian universities offering nursing and health informatics programs, provided valuable implementation support, evaluation capacity, and sustainability. A comparison of contextual factors influencing automation implementation success across settings is summarized in Table 3.

**TABLE 3:** Contextual factors influencing automation implementation success.

Factor	High‐resource settings	Low‐resource settings (like Rafidia)	Implications for implementation
Infrastructure	Reliable power, high‐speed internet	Intermittent power (reported in 78% of facilities), limited bandwidth	Need for offline functionality, backup systems, gradual rollout
Financial resources	Dedicated technology budgets, vendor support	Constrained operational budgets, import restrictions	Prioritize low‐cost solutions, seek donor/NGO partnerships
Workforce readiness	High digital literacy, dedicated IT staff	Limited prior exposure, high staff turnover	Extensive training needed, peer mentorship models
Regulatory environment	Clear liability frameworks, data protection laws	Evolving regulations, limited enforcement	Develop institutional policies, ethical oversight committees
Cultural factors	Individual decision‐making, technology acceptance	Family‐centered care, relational priorities	Preserve human interaction, involve families in design
Political/geopolitical	Stable geopolitical environment, open technology markets	Political instability, restricted access to technology markets	Design for intermittent connectivity, import flexibility, emphasize data security

#### 3.1.4. Theme 4: Ethical, Legal, and Cultural Considerations

##### 3.1.4.1. Data Privacy and Security

Data privacy emerged as a paramount ethical concern, particularly acute in politically unstable environments [61]. Emergency nurses and patients expressed concerns about the security of health data stored in electronic systems potentially vulnerable to cyberattacks, unauthorized access, or government surveillance. In the Palestinian context, concerns about data security were heightened by political sensitivities and documented instances of health data breaches [62].

##### 3.1.4.2. Equity in Access to Automated Care

Equity considerations centered on ensuring that automation benefits did not disproportionately favor certain patient populations or create disparities in care quality [63]. Research documented that AI algorithms trained predominantly on data from high‐income countries sometimes performed poorly for patients from different ethnic, linguistic, or socioeconomic backgrounds [64].

Within Palestinian EDs serving mixed populations, including refugees, rural communities, and urban residents, ensuring equitable automation performance required intentional algorithm validation across diverse groups and continuous monitoring for disparate outcomes.

##### 3.1.4.3. Maintaining Human Touch

The preservation of compassionate, person‐centered care in technologically augmented emergency nursing emerged as a critical ethical consideration [65]. Qualitative studies with patients and families revealed strong preferences for human interaction during emergency care episodes, with concerns that automation might depersonalize care or reduce nurse presence [66].

Cultural considerations in Arab societies, where family involvement in healthcare decisions and personal relationships with providers are highly valued, suggested that automation implementation must be carefully balanced with preservation of interpersonal care dimensions [67].

#### 3.1.5. Theme 5: Rafidia Hospital as a Case Context

##### 3.1.5.1. Analysis of Existing Pilot Projects

Gray literature from Rafidia Governmental Hospital, in collaboration with the Palestinian Ministry of Health, documented two primary automation pilot initiatives launched between 2022 and 2024 [68]. The first involved the implementation of an AI‐assisted triage tool developed in partnership with the Palestinian Ministry of Health, designed to support initial acuity assessment in the ED. Preliminary evaluation using a pre/post design with survey feedback indicated improved triage consistency, with inter‐rater reliability increasing from 0.68 to 0.81 following system introduction. While the gray literature did not report on statistical significance, this increase represents a notable improvement in clinical agreement.

The second pilot examined voice‐to‐text documentation technology for nurse charting, aiming to reduce documentation burden. Participating nurses reported time savings averaging 25 min per shift in postimplementation surveys, though system accuracy for Arabic medical terminology required ongoing refinement.

**FIGURE 2:**
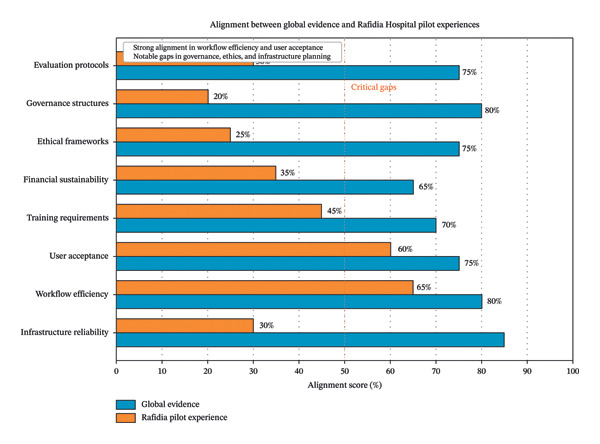
Alignment between global evidence and Rafidia Governmental Hospital’s pilot experiences in automation integration. The figure illustrates five thematic domains derived from the integrative review synthesis: (1) automation technologies implemented (AI‐assisted triage support and voice‐to‐text documentation); (2) outcomes achieved in pilot studies (improved triage consistency and documentation time savings); (3) contextual barriers encountered (power interruptions, connectivity limitations, and language adaptation challenges); (4) facilitators identified (nurse involvement, phased approach, and Ministry of Health collaboration); and (5) identified gaps requiring attention (governance frameworks, evaluation infrastructure, training capacity, sustainability planning, and technical support). Arrows indicate the directional relationship between global evidence themes and local pilot findings, highlighting areas of convergence and divergence relevant to implementation planning in resource‐limited, conflict‐affected settings.

Both pilots highlighted the critical importance of reliable electricity and internet connectivity; power interruptions occurring 2–3 times weekly necessitated manual backup systems and resulted in data loss incidents. The alignment between global evidence and Rafidia’s pilot experiences is summarized in Figure 2, created using Microsoft PowerPoint for layout and Adobe Illustrator for final graphic refinement.

##### 3.1.5.2. Identified Gaps Between Evidence and Current Capabilities

Despite progress, significant gaps remained between evidence‐based best practices and current capabilities at Rafidia:

1. Governance and policy: Absence of institutional protocols for automation governance, data management policies aligned with emerging Palestinian health data regulations, and liability frameworks for AI‐assisted clinical decisions.
2. Evaluation infrastructure: Limited systematic evaluation frameworks for pilot projects, including patient outcomes, cost‐effectiveness, and nurse satisfaction metrics.
3. Training capacity: Underdeveloped training infrastructure and identified needs for ongoing competency development, simulation facilities for practicing with automation during emergencies, and technical support capacity within the hospital.
4. Sustainability planning: Reliance on pilot funding from academic partners and international NGOs rather than sustainable operational budgets, raising concerns about long‐term maintenance and scaling.
5. Technical support capacity: Limited in‐house expertise to troubleshoot system failures, perform regular maintenance, and adapt systems to evolving clinical needs, creating dependency on external vendors.

## 4. Discussion

This integrative review demonstrates that automation technologies hold substantial promise for enhancing efficiency, safety, and quality in emergency nursing practice; however, successful integration requires careful adaptation to infrastructural realities, human resource capacities, and cultural contexts of implementation settings [69]. The evidence synthesis reveals that automation is not a uniform solution but rather a diverse portfolio of technologies with varying applicability, implementation requirements, and outcome profiles across different contexts [70]. Documentation automation, with its lower implementation complexity and consistent time‐saving benefits, demonstrated the greatest contextual adaptability for low‐resource settings, while advanced robotics and highly complex AI systems appear less suitable without substantial infrastructural investment.

Documentation automation and triage support systems consistently yield positive outcomes with manageable implementation barriers, positioning them as priority technologies for low‐resource settings [71]. In contrast, advanced robotics and complex AI‐driven systems require substantial resources and technical dependencies that likely exceed current capacities at hospitals like Rafidia.

Rafidia Hospital’s pilot experiences mirror global trends while highlighting challenges unique to the Palestinian context, including power interruptions and restricted access to technical support due to import limitations [72]. Geopolitical instability disrupts not only infrastructure but also access to technology markets, support networks, and sustainable funding. Such resource fluctuations—including unpredictable budgets, supply chain disruptions, and workforce mobility—create implementation uncertainties that automation literature, developed largely in stable environments, fails to address [73].

Our findings contrast with generic implementation models by emphasizing that in resource‐limited settings, the sequence and pace of implementation must be fundamentally different. Where high‐resource settings can implement comprehensive systems simultaneously, our analysis suggests that for settings like Rafidia, success depends on starting with discrete, high‐impact modules and building capacity incrementally.

### 4.1. Implications for Practice at Rafidia Hospital

Based on the synthesis of evidence and contextual analysis, several practice implications emerge for Rafidia Hospital’s ED.

#### 4.1.1. Prioritization of Low‐Technology, High‐Impact Automation

Enhancing documentation support systems, potentially building upon the existing voice‐to‐text pilot, offers significant time savings with manageable implementation requirements. Focusing on Arabic language optimization, improving offline functionality, and expanding to additional documentation types (patient education and handoff communication) could maximize return on existing investment. This could involve deploying tablet‐based documentation systems with local server synchronization to address connectivity gaps.

#### 4.1.2. Development of Hybrid Decision‐Support Models

Triage support systems that present evidence‐based recommendations while requiring nurse validation and allowing override based on clinical judgment exemplify a balanced approach that addresses both efficiency goals and concerns about deskilling and serves as a key strategy to mitigate automation bias [74]. Similarly, automated medication verification that prompts nurse attention to potential errors while permitting informed override preserves professional autonomy while enhancing safety. This hybrid approach aligns with both global evidence and cultural preferences for maintaining clinician oversight.

#### 4.1.3. Investment in Continuous, Simulation‐Based Training

Training programs should encompass technical skills (system operation and troubleshooting), cognitive skills (interpreting automated recommendations and recognizing system limitations), and adaptive skills (workflow integration and patient communication about automation use) [75]. Simulation scenarios addressing automation failures and emergency manual backup procedures ensure preparedness for technical disruptions. This could involve quarterly in situ simulations focusing on system failure scenarios and manual reversion procedures.

#### 4.1.4. Formation of Multidisciplinary Implementation Teams

A multidisciplinary automation implementation team including emergency nurses, physicians, information technology (IT) staff, quality improvement specialists, and patient representatives would provide essential governance and oversight [76]. This team should guide technology selection, implementation planning, ongoing evaluation, and iterative system refinement based on user feedback and outcome data. Including patient representatives is particularly important in the Arab cultural context where family‐centered care is valued. Patient representatives could be recruited through existing hospital patient or family advisory councils, where these exist, or through community outreach.

#### 4.1.5. Proposed Implementation Framework for Rafidia Hospital

A phased, evidence‐informed implementation framework tailored to Rafidia Hospital’s context and resources is proposed below. The timeline allocations are based on typical implementation cycles in similar resource‐limited settings, with longer phases for readiness and evaluation to accommodate infrastructure limitations and the need for iterative adaptation.

##### 4.1.5.1. Phase 1: Readiness Assessment and Staff Engagement (Months 1–3)

This extended 3‐month phase allows for thorough assessment of both technical and human factors, which is critical in settings with infrastructure limitations.

• Conduct a comprehensive needs assessment using a combination of validated tools (e.g., NASA‐TLX for workload), infrastructure audit checklists, and focus group guides, led by the Automation Steering Committee.
• Assess current infrastructure capabilities including power reliability, internet bandwidth, and IT support capacity.
• Establish an Automation Steering Committee with representation from emergency nurses, physicians, IT personnel, administration, and patient representatives.
• Engage staff through transparent communication about automation goals, expected benefits, and mechanisms for ongoing input.

##### 4.1.5.2. Phase 2: Pilot Implementation (Months 4–9)

The 6‐month pilot duration accommodates the learning curve in settings with limited prior digital health experience and allows for troubleshooting of infrastructure issues.

• Select a single, high‐priority automation module based on needs assessment findings. Based on this review’s findings, documentation automation emerges as a strong, evidence‐supported candidate for a starting point in resource‐limited settings, though the final choice must be locally determined.
• Implement pilot in a bounded ED area or patient population.
• Provide intensive training including initial skill building, hands‐on practice, and just‐in‐time coaching.
• Establish redundancy and backup systems to ensure care continuity if automation fails.

##### 4.1.5.3. Phase 3: Evaluation, Adaptation, and Scaling (Months 10–15)

This 6‐month evaluation period enables comprehensive assessment and system refinement before scaling decisions.

• Analyze pilot data against predetermined success criteria (efficiency, safety, and user satisfaction).
• Conduct structured debriefing with pilot participants to understand implementation challenges.
• Based on evaluation findings, refine the automation system including technical adjustments and workflow modifications.
• If the pilot demonstrates success, develop a scaling plan for department‐wide implementation.

##### 4.1.5.4. Phase 4: Integration and Sustainability (Months 16–24 and Ongoing)

This open‐ended phase focuses on institutionalization, recognizing that sustainability requires ongoing attention and resources.

• Integrate automation into standard operating procedures, orientation programs, and continuous competency assessments.
• Explore opportunities to incorporate automation content into Palestinian nursing curricula through collaboration with universities.
• Establish systematic evaluation processes including regular outcome monitoring and annual comprehensive reviews.
• Transition from pilot funding to sustainable operational budget integration.

#### 4.1.6. Unique Contribution to Nursing Management Scholarship

Beyond its contextual application to a Palestinian hospital, this review makes a unique contribution to nursing management scholarship by systematically deconstructing the “implementation paradox” of healthcare automation, whereby automation and AI technologies proven effective in high‐resource settings often underperform in resource‐limited environments, not due to technological inadequacy but owing to a fundamental mismatch between the implicit assumptions embedded in mainstream implementation models and the operational realities of settings like Rafidia Hospital. By foregrounding infrastructure volatility, workforce digital literacy, and geopolitical fragility as core implementation determinants, this review offers nursing leaders a transferable analytical framework and a phased implementation logic that prioritizes resilience and sustainability. This reframing shifts the discourse from passive technology transfer toward context‐informed innovation, empowering nurse managers to function as active architects of locally appropriate technological change.

## 5. Limitations of the Review

Several limitations warrant consideration when interpreting this review’s findings. First, the limited availability of published research specifically from Palestinian healthcare settings necessitated reliance on contextual extrapolation from studies conducted in other low‐resource and conflict‐affected environments [77]. While these settings share important similarities with Palestine, contextual nuances may limit direct applicability of some findings. We attempted to mitigate this limitation by actively including gray literature from Rafidia Hospital and prioritizing studies from regional Middle Eastern contexts where available.

Second, gray literature from Rafidia Hospital, while providing valuable contextual insights, lacks the methodological rigor of peer‐reviewed research [78]. Pilot project evaluations were primarily descriptive and lacked comparison groups, limiting causal inferences about automation impacts.

Third, the rapidly evolving nature of automation technology means that some findings may become outdated quickly. This review captured literature up to March 2025; technologies emerging after this date, such as newer generative AI applications for clinical documentation, are not reflected in this synthesis.

Fourth, this review focused primarily on technological and operational dimensions of automation, with less attention to detailed economic analyses including comprehensive cost‐effectiveness evaluations [79].

Finally, regarding generalizability, while the proposed framework is tailored to Rafidia Hospital, its core principles, phased implementation, nurse‐centered design, and infrastructure‐conscious planning are likely transferable to similar low‐resource governmental hospitals. However, the specific technology choices and timeline would require adaptation to local infrastructure, workforce, and cultural contexts. The framework may also need adaptation for private sector hospitals in Palestine, which may have different resource profiles, operational constraints, and patient populations.

## 6. Conclusion and Recommendations

### 6.1. Summary of Evidence

This integrative review synthesized diverse evidence regarding automation integration in emergency nursing, with specific application to Rafidia Governmental Hospital in Palestine. The evidence demonstrates that automation technologies can enhance emergency nursing efficiency, reduce errors, and support clinical decision‐making when implemented thoughtfully [80]. However, success is highly contingent upon contextual adaptation, nurse‐centered design processes, and sustainable resourcing that accounts for infrastructure limitations and workforce needs.

Rafidia Hospital’s position as a governmental facility in a politically complex, resource‐limited environment necessitates implementation strategies that differ substantially from those employed in high‐resource settings. Pragmatic prioritization of high‐impact, lower‐complexity automation; phased implementation with continuous evaluation; robust training and support systems; and careful attention to ethical, cultural, and equity considerations emerge as critical success factors.

### 6.2. Recommendations

#### 6.2.1. For Rafidia Hospital

1. Establish a multidisciplinary automation task force with a clear mandate, resources, and accountability for guiding ED automation initiatives.
2. Prioritize infrastructure strengthening including backup power systems, enhanced internet connectivity, and IT support capacity as foundational prerequisites for sustainable automation.
3. Implement the proposed phased framework beginning with documentation automation, emphasizing iterative learning and staff engagement throughout the process.
4. Foster nurse‐led innovation by supporting frontline nurse participation in automation design, implementation, and evaluation.

#### 6.2.2. For Researchers

1. Conduct longitudinal, context‐sensitive studies using mixed methods designs, examining automation outcomes in Palestinian and similar emergency care settings.
2. Prioritize implementation science research exploring adaptation strategies, sustainability mechanisms, and scaling pathways for automation in resource‐limited environments using participatory action research or stepped‐wedge cluster randomized trials.
3. Investigate cultural dimensions of automation acceptance and appropriate adaptation for Arab healthcare contexts.
4. Examine equity implications of automation adoption, particularly impacts on vulnerable populations and potential disparities.

#### 6.2.3. For Policymakers

1. Develop clear, supportive regulations for health technology integration in Palestinian governmental hospitals, addressing liability frameworks for AI‐assisted decisions and data protection standards that are proportionate to the Palestinian context, avoiding overly burdensome requirements that may be unattainable.
2. Allocate dedicated funding for health technology innovation, including pilot projects, implementation support, training programs, and ongoing maintenance.
3. Invest in foundational infrastructure strengthening across governmental hospitals to enable technology adoption.
4. Facilitate regional collaboration and knowledge exchange through support for hospital networks and academic‐practice partnerships.

### 6.3. Future Directions

The evolving landscape of healthcare automation presents both opportunities and imperatives for continued research and practice development. Implementation science studies specifically focused on automation scalability in conflict‐affected and resource‐limited settings would address critical knowledge gaps. Such research should examine not only technical implementation but also sustainability mechanisms, workforce adaptation, and patient outcome impacts across diverse contexts. Future studies should employ participatory action research designs to codevelop and evaluate automation with frontline nurses in Palestine.

Cross‐hospital collaborations within Palestine offer potential to build shared evidence bases, pool resources, and accelerate learning. A coordinated West Bank emergency nursing automation network could facilitate technology sharing, joint procurement reducing costs, collaborative evaluation strengthening evidence quality, and peer mentoring supporting implementation.

Ultimately, the goal is not automation for its own sake but rather the enhancement of emergency nursing practice to better serve Palestinian communities. Technology should be viewed as one tool among many for supporting nurses to provide safe, effective, compassionate care under challenging circumstances. Success will be measured not by the sophistication of technologies adopted but by meaningful improvements in nurse well‐being, patient outcomes, and the resilience of Palestinian emergency care systems, a testament to the nursing profession’s core values of caring, compassion, and human presence, enhanced but never replaced by technology.

**Nomenclature**
AI: Artificial intelligence
ED: Emergency department
HIT: Health information technology
IMEMR: Index Medicus for the Eastern Mediterranean Region
IT: Information technology
MMAT: Mixed Methods Appraisal Tool
NGO: Nongovernmental organization
PRISMA: Preferred Reporting Items for Systematic Reviews and Meta‐Analyses
RPA: Robotic process automation
WHO: World Health Organization

## Author Contributions

Ibrahim Aqtam: conceptualization, methodology, investigation, data curation, formal analysis, writing–original draft, and writing–review and editing.

Mustafa Shouli: conceptualization, methodology, project administration, supervision, validation, and writing–review and editing.

Saqr Alkorom: investigation, data curation, validation, and writing–review and editing.

## Funding

No external funding was received for this research.

## Ethics Statement

This integrative review was conducted in accordance with the ethical standards for research. Since this study synthesizes previously published literature and does not involve direct data collection from human subjects, formal ethics approval and individual consent were not required. All included studies were reviewed in compliance with academic integrity and citation standards.

## Consent

Please see the Ethics Statement.

## Conflicts of Interest

The authors declare no conflicts of interest.

## Supporting Information

Additional supporting information can be found online in the Supporting Information section.

## Supporting information


**Supporting Information 1** Supporting File 1: PRISMA 2020 Checklist. This file provides the completed PRISMA 2020 checklist confirming adherence to reporting standards for systematic and integrative reviews. Each checklist item is mapped to the corresponding page number in the manuscript where the relevant information is reported, covering all sections including title, abstract, introduction, methods, results, and discussion.

**Supporting Information 2** Supporting File 2: Quality appraisal summary table. This file contains a comprehensive quality appraisal summary for all 47 included sources. Part A presents the Mixed Methods Appraisal Tool (MMAT) scores and ratings for the 28 included empirical studies, including individual criteria met, percentage scores, overall quality ratings, and key methodological limitations. Part B presents quality appraisal results for gray literature sources using an adapted AACODS checklist, assessing authority, accuracy, coverage, objectivity, date, and significance of each source.

## Data Availability

No new primary data were generated or analyzed in this integrative review. All data are derived from previously published sources cited in the reference list.
