# Supplementary material for: An Integrative Review of Automation Integration in Emergency Nursing Practice: Evidence Synthesis and Contextual Application to Rafidia Governmental Hospital, Palestine
Source: J Nurs Manag. 2026 Apr 12;2026:2678581. doi: 10.1155/jonm/2678581 (PMC13071338; doi:10.1155/jonm/2678581)
Supplement: Supplementary file 2 — Supporting Information 2 Supporting File 2: Quality appraisal summary table. This file contains a comprehensive quality appraisal summary for all 47 included sources. Part A presents the Mixed Methods Appraisal Tool (MMAT) scores and ratings for the 28 included empirical studies, including individual criteria met, percentage scores, overall quality ratings, and key methodological limitations. Part B presents quality appraisal results for gray literature sources using an adapted AACODS checklist, assessing authority, accuracy, coverage, objectivity, date, and significance of each source. [file JONM-2026-2678581-s001.docx]

**Quality Appraisal Summary Table**

**Part A: Quality Appraisal of Included Empirical Studies (n=28) Using MMAT**

| **Study ID / Reference** | **Study Design** | **MMAT Criteria Met** | **MMAT Score (%)** | **Quality Rating** | **Key Methodological Limitations** |
| --- | --- | --- | --- | --- | --- |
| **High-Income Country Studies** |  |  |  |  |  |
| Levin et al. (2018) [24] | Quantitative (non-randomized) | 4/5 | 80% | Moderate | Single site, lack of blinding |
| Raita et al. (2019) [25] | Quantitative (non-randomized) | 5/5 | 100% | High | Well-conducted, but retrospective design |
| Hatton et al. (2025) [26] | Quantitative (RCT) | 5/5 | 100% | High | Rigorous RCT design |
| Goss et al. (2016) [29] | Quantitative (descriptive) | 3/5 | 60% | Moderate | Small sample, single site |
| Ebbers et al. (2022) [30] | Quantitative (retrospective) | 4/5 | 80% | Moderate | Retrospective design, potential confounding |
| Campanella et al. (2016) [31] | Systematic review & meta-analysis | 5/5 | 100% | High | Comprehensive search, clear methods |
| Holmgren et al. (2022) [34] | Quantitative (observational) | 4/5 | 80% | Moderate | Observational design, potential selection bias |
| Arndt et al. (2017) [35] | Mixed methods | 5/5 | 100% | High | Strong integration of quantitative and qualitative data |
| McHugh et al. (2012) [36] | Quantitative (cross-sectional) | 4/5 | 80% | Moderate | Cross-sectional design limits causal inference |
| Shanafelt et al. (2019) [37] | Quantitative (cross-sectional) | 5/5 | 100% | High | Large sample, validated measures |
| Robertson et al. (2017) [38] | Quantitative (cross-sectional) | 4/5 | 80% | Moderate | Self-report data, potential response bias |
| Sinsky et al. (2019) [39] | Qualitative | 4/5 | 80% | Moderate | Rich qualitative data, but limited transferability |
| Gardner et al. (2019) [40] | Quantitative (cross-sectional) | 5/5 | 100% | High | Large sample, rigorous analysis |
| Cresswell et al. (2013) [41] | Qualitative | 5/5 | 100% | High | In-depth interviews, strong theoretical framing |
| Kaushal et al. (2003) [42] | Systematic review | 4/5 | 80% | Moderate | Older review, but rigorous methods for its time |
| Nuckols et al. (2014) [43] | Systematic review & meta-analysis | 5/5 | 100% | High | Comprehensive, well-conducted meta-analysis |
| Smith et al. (2013) [44] | Quantitative (cohort) | 5/5 | 100% | High | Large cohort, robust validation |
| Lyell & Coiera (2017) [45] | Systematic review | 5/5 | 100% | High | Rigorous systematic review methods |
| Goddard et al. (2012) [46] | Systematic review | 5/5 | 100% | High | Comprehensive review of automation bias |
| Bonafide et al. (2017) [47] | Quantitative (observational) | 4/5 | 80% | Moderate | Innovative video analysis, but single site |
| Winters et al. (2018) [48] | Systematic review | 5/5 | 100% | High | High-quality systematic review |
| **Middle-Income Country Studies** |  |  |  |  |  |
| Adler et al. (2019) [27] | Quantitative (validation study) | 4/5 | 80% | Moderate | Multi-site validation, but specific population |
| Bardhia et al. (2025) [28] | Quantitative (cross-sectional) | 3/5 | 60% | Moderate | Cross-sectional design, single site |
| Syzdykova et al. (2017) [32] | Systematic review | 5/5 | 100% | High | Focus on low-resource settings, comprehensive |
| Stevenson et al. (2018) [33] | Qualitative | 4/5 | 80% | Moderate | Rich qualitative data, but limited context |
| Zayyad & Toycan (2018) [53] | Quantitative (cross-sectional) | 3/5 | 60% | Moderate | Exploratory survey, limited generalizability |
| Biruk et al. (2014) [55] | Quantitative (cross-sectional) | 4/5 | 80% | Moderate | Adequate sample, but single country |
| Kruse et al. (2016) [56] | Systematic review | 5/5 | 100% | High | Comprehensive review of barriers |
| Fares et al. (2014) [73] | Descriptive/case study | 3/5 | 60% | Moderate | Descriptive, limited empirical data |
| **Low-Income/Conflict Settings Studies** |  |  |  |  |  |
| Kiberu et al. (2017) [51] | Literature review | 4/5 | 80% | Moderate | Focused on Uganda, rigorous for review type |
| Qaraman et al. (2022) [10] | Quantitative (cross-sectional) | 3/5 | 60% | Moderate | Single site, self-report data |
| Shalash et al. (2024) [62] | Qualitative | 4/5 | 80% | Moderate | Rich context-specific data, limited transferability |
| Mowafi et al. (2020) [17] | Descriptive/commentary | 3/5 | 60% | Moderate | Expert opinion, limited empirical data |
| Giacaman et al. (2011) [72] | Mixed methods | 4/5 | 80% | Moderate | Strong contextual analysis, but descriptive |

**Part B: Quality Appraisal of Included Systematic Reviews (n=8) Using MMAT**

| **Study ID / Reference** | **MMAT Criteria Met** | **MMAT Score (%)** | **Quality Rating** | **Key Strengths/Limitations** |
| --- | --- | --- | --- | --- |
| Morley et al. (2018) [1] | 5/5 | 100% | High | Comprehensive search, clear inclusion criteria |
| Topol (2019) [2] | 4/5 | 80% | Moderate | Narrative review, not fully systematic |
| Bates et al. (2021) [4] | 5/5 | 100% | High | Rigorous scoping review methodology |
| Harerimana et al. (2021) [5] | 5/5 | 100% | High | Well-conducted scoping review |
| Cresswell & Sheikh (2013) [6] | 5/5 | 100% | High | Strong interpretive review methods |
| Greenhalgh et al. (2017) [7] | 5/5 | 100% | High | Developed NASSS framework, rigorous |
| Shaw et al. (2019) [13] | 4/5 | 80% | Moderate | Focused on implementation, but selective |
| Sutton et al. (2020) [74] | 5/5 | 100% | High | Comprehensive overview of CDSS |

**Part C: Quality Appraisal of Included Case Studies (n=6) Using MMAT (Adapted)**

| **Study ID / Reference** | **Clear Research Questions?** | **Appropriate Data Collection?** | **Rich Description?** | **Transferable Findings?** | **Overall Quality Rating** |
| --- | --- | --- | --- | --- | --- |
| Dowding et al. (2015) [3] | Yes | Yes | Yes | Partial | **High** |
| Ford et al. (2006) [49] | Yes | Yes | Yes | Yes | **High** |
| Sittig & Singh (2010) [50] | Yes | N/A (theoretical) | Yes | Yes | **High** (theoretical contribution) |
| Flanagan & Marsh (2000) [57] | Partial | Yes | Yes | Partial | **Moderate** |
| Øvretveit et al. (2007) [76] | Yes | N/A (framework) | Yes | Yes | **High** |
| Cook et al. (2013) [75] | Yes | Yes (systematic review) | Yes | Yes | **High** |

**Part D: Quality Appraisal of Grey Literature Sources (n=5) Using Adapted AACODS Checklist**

| **Document Source** | **Authority** | **Accuracy** | **Coverage** | **Objectivity** | **Date** | **Significance** | **Overall Quality Rating** |
| --- | --- | --- | --- | --- | --- | --- | --- |
| Palestinian Ministry of Health (2023) [8] | High (government) | High | Comprehensive | High (policy document) | Current | High | **High** |
| Palestinian Ministry of Health (2023) [11] | High (government) | High | Focused on pilots | High | Current | High | **High** |
| WHO (2023) [52] | Very High (international agency) | High | Regional overview | High | Current | Very High | **High** |
| Palestine Economic Policy Research Institute (2022) [54] | High (research institute) | High | Economic focus | High | Current | Moderate | **High** |
| Rafidia Hospital & MOH (2024) [68] | High (hospital + ministry) | Moderate (preliminary data) | Focused on two pilots | High | Current | Very High (context-specific) | **Moderate** (due to preliminary evaluation methods) |

**Summary of Quality Appraisal Findings**

| **Source Type** | **Number of Sources** | **Mean Quality Score** | **Quality Range** |
| --- | --- | --- | --- |
| Empirical Studies (High-Income) | 21 | 89% | 60–100% |
| Empirical Studies (Middle-Income) | 7 | 77% | 60–100% |
| Empirical Studies (Low-Income/Conflict) | 4 | 70% | 60–80% |
| Systematic Reviews | 8 | 95% | 80–100% |
| Case Studies | 6 | N/A (qualitative) | Moderate to High |
| Grey Literature | 5 | N/A (qualitative) | Moderate to High |
| **Overall** | **51*** | **84% (empirical only)** | **60–100%** |

***Note:****Total exceeds 47 because some sources are counted in multiple categories (e.g., a systematic review may also be an empirical study if it includes meta-analysis).*

**Interpretation of Quality Ratings**

| **Quality Rating** | **Definition** |
| --- | --- |
| **High (80–100%)** | Study meets most or all MMAT criteria; minimal methodological limitations; findings are trustworthy and well-supported. |
| **Moderate (60–79%)** | Study meets some MMAT criteria; some methodological limitations exist but do not fundamentally undermine findings. |
| **Low (<60%)** | Study meets few MMAT criteria; significant methodological limitations; findings should be interpreted with caution. |
